# Supplementary material for: Changes in Essential Fatty Acids and Ileal Genes Associated with Metabolizing Enzymes and Fatty Acid Transporters in Rodent Models of Cystic Fibrosis
Source: Int J Mol Sci. 2023 Apr 13;24(8):7194. doi: 10.3390/ijms24087194 (PMC10138779; doi:10.3390/ijms24087194)
Supplement: Supplementary file 1 [file ijms-24-07194-s001.zip › ijms-2317268-supplementary.pdf]

Table S1: Dietary fatty acids in the normal and high fat rodent chow and 50:50 mixture.

**Fatty acids**	**% in normal fat diet (Teklad Global Soy Protein-Free Extruded Rodent Diet)**	**% in high fat diet (Teklad Global 19% Protein Extruded Rodent Diet)**	**% in 50:50 mixture**
C16:0 Palmitic	0.60	0.90	0.75
C18:0 Stearic	0.10	0.20	0.15
C18:1ω9 Oleic	1.10	1.70	1.40
C18:2ω6 Linoleic (LA)	2.60	3.90	3.25
C18:3ω3 Linolenic (ALA)	0.30	0.40	0.35
Ratio LA/ALA	8.7:1	9.75:1	9.3:1
Total saturated	0.80	1.20	1.00
Total monounsaturated	1.10	1.70	1.40
Total polyunsaturated	2.90	4.40	3.65
Calories from Fat	16	22	19
Fat	6.5	9	7.75

Table S2: qPCR primer sequences

Gene	Forward and reverse primer sequences (5' to 3')
β-actin	CTATGAGCTGCCTGACGGTC<br>AGTTTCATGGATGCCACAGG
Cyclophilin A	GCAGACATGGTCAACCCCACCG<br>TGGAACTTTGTCTGCAAACAGCTCG
Cftr	AAGCTGAAAGCAGGTGGGAT<br>TGCTCCGACCACAATGAACA
Elovl2	CTTGTGGTCAAAGCTTCTTC<br>GAGGTATTTCTTCCACCAAAG
Elovl	TTCTTCGTAAGAACAACCAC<br>ATAGTACGAGTACATGAGGAC
FATP1 (Scl27a1)	GACAGTGTATCATCTACGGG<br>CATATTTCACCGATGTACTGC
FATP2 (*Scl27a2)*	GGGACTGGTAGATTTTGTTG<br>GTATCTTGTATTCTCAGGAACC
FATP4 (Scl27a4)	CAAGGGTTTTACAGATAAGCTC<br>GGAATCCATAGTACACCAGG
Fads1	GTACTTCTTCCTGATTGGAC<br>GTAAGTGAAGAAGACACGAAC
Fads2	CTTCTTCAATGACTGGTTCAG<br>CTTCAGTGAACTCACAATGTC
Faah	TCCAAAACTTCAAAGGTGAC<br>GATACATCTCAATCTCATGCTG
Got2	CAAAGAATACCTACCCATCG<br>AAATCTTTGCAGAAAGCTGG
Cd36	AAGGAATTTGTCCTATTGGG<br>GAGACTTCTCAACAAAAGGTG
Col1a1	TGGTTTGGAGAGAGCATGACCGAT<br>TTGGTCCATGTAGGCTACGCTGTT
Col3a1	TTTCAAGATCAACACTGAGG<br>TATTCTCCGCTCTTGAGTTC
